# Research on Spatiotemporal Association between Tourism and Transportation Based on CGS Model

**DOI:** 10.1155/2022/9559170

**Published:** 2022-03-02

**Authors:** Yanan Xu, Jianxin Qin, Tao Wu, Bo Liu, Yuanping Xia

**Affiliations:** ^1^Hunan Key Laboratory of Geospatial Big Data Mining and Application, Hunan Normal University, Changsha 410081, Hunan, China; ^2^Faculty of Geomatics, East China University of Technology, Nanchang 330013, Jiangxi, China

## Abstract

Tourism and transportation generally have an inseparable association. However, there are still many limitations in the existing research on it. For example, most scholars only adopt one single model method, which fails to consider geospatial elements. Moreover, some researchers simply use socioeconomic data for analysis and research and ignore the solid spatial characteristics between tourism and transportation, which leads to deviations in the results. To solve these problems, this article proposed a spatiotemporal association model by comprehensively using coupling coordination degree, gravity center model, and spatial coincidence degree. Based on the tourism economic and attraction spatial data, and the transportation and its network spatial data, the association between tourism and transportation can be revealed by the proposed model. This study conducted a quantitative analysis on the tourism and transportation industry in Jiangxi Province, China, from 2005 to 2019, and the results show that: (1) the coupling coordination degree of tourism and transportation increases year by year; (2) the change in gravity center of tourism and transportation is subtle. The mean value of spatial overlap is 80.33 km, while the mean value of inter-annual variation consistency is 0.56; (3) the spatial coincidence degree of tourism and transportation in Jiangxi Province indicates a steady upward trend and reaches 0.78 in 2019; and (4) based on the evolution trend in the coupling coordination degree, gravity center coupling model, and spatial coincidence degree of tourism and transportation, it can be seen that the slopes of their trend functions are similar and consistent—the slopes are 0.0239, 0.0253, and 0.0319, respectively—and the standard deviation of the slopes of the three is only 0.000018.

## 1. Introduction

It is well known that tourism has already become the most active power to enhance regional economic development and create job opportunities. As a primary leading industry of economic development, the transportation industry is an essential prerequisite for tourism. Convenient transportation will help tourists make better choices about the tourist destinations and travel schedules and improve the quality and satisfaction throughout the trip. Thus, studying the association between transportation and tourism will be of great practical significance to develop and integrate tourism resources and improve transportation service quality [1].

A wealth of research has been conducted on the association between transportation and tourism. Lundgren et al. proposed a way to grasp the relative positioning of tourist destinations within the geographical scope of the overall tourism level [2]. Jameel et al. studied how transportation affects destination development, which was a part of classic demand for international tourism functions [3]. David reviewed the prominent intersections of tourism and transport and made predictions for future directions [4]. Adeleke identified a number of factors that influenced the development of transportation and tourism in Nigeria and found that variables contribute 60.4% to the development of tourism infrastructure [5]. According to KhadarooJ's survey, visitors from Europe, America, and Asia were particularly sensitive to the research area's transportation [3]. Based on the concept of physical coupling, Deng et al. constructed the coordination degree model of tourism, transport, and low-carbon city coupling. He analyzed the low-carbon city work in Chongqing from 2008 to 2017 from the aspects of the coupling and evolution trend [6]. Gao et al. used Panel data of Chinese cities from 2004 to 2015 to evaluate the impact of high-speed rail on tourism growth [7]. Chen et al. applied a complex network theory, social network analysis, kernel density analysis, binary auto-association, and other methods for analyzing complex transportation and tourism potential [8]. Based on the gravity center and coupling coordination model, Lu et al. explored the gravity center coupling dynamics and coordinated development degree of tourism and transportation [9]. Bao et al. believed that the convenience of transportation was an important symbol to measure tourism development [10]. With the help of the DEA-Malmquist model, natural breakpoint method, and restricted dependent variable model, Wang et al. explored the spatiotemporal interaction between transportation and tourism development efficiency and their influencing mechanism [11, 12]. Wang and Chen proposed a PVAR model, in which he empirically analyzed the interaction between transportation and tourism, as well as the long-term and short-term dynamic impact [13]. Many other scholars have also studied the association between transportation and tourism from different perspectives [14–16].

Although many studies focus on the relationship between tourism and transport, some problems still exist, such as the lack of systematicness in research methods. Many scholars use a single model to study the relationship between tourism and transportation. For example, some researchers only use socioeconomic data for analysis and research without considering the geospatial elements. However, tourism and transportation have prominent spatial characteristics. Moreover, the single selection calculation method is inefficient when determining the weight. Aiming at solving the problems mentioned above, this study utilized coupling coordination degree, the center of gravity model, and spatial coincidence degree (henceforth referred to as CGS) to analyze the association between tourism and transportation. The study also selects tourism economic and attraction spatial data and transportation and its network spatial data from 2005 to 2019 in Jiangxi Province, China, to conduct specific case analysis.

## 2. Methodology

**Figure 1:**
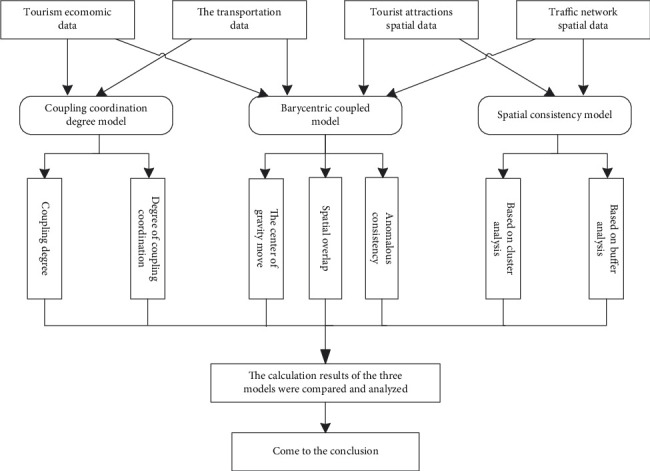
CGS spatiotemporal correlation model framework.

The operation framework of the spatiotemporal correlation model proposed in this article is shown in Figure 1. This model comprehensively uses tourism economic data, transportation data, scenic spot spatial data, and traffic network spatial data. It can reveal the correlation between tourism and traffic by calculating the coupling coordination degree, the center of gravity model, and spatial coincidence degree between various data.

### 2.1. Coupling Coordination Degree

Transportation and tourism economic development are two systems that interact and promote each other. By referring to the coupling concept and principle [17], the coupling degree between tourism and transportation development level can be defined as (1) [18],

(1) $$\begin{array}{c} C=\frac{T_{1}\times T_{2}}{{\left(\alpha \times T_{1}+\beta \times T_{2}\right)}^{2}}, \end{array}$$

where *T*_1_ is the comprehensive evaluation index of the tourism economy in a specific region and *T*_2_ is the comprehensive evaluation index of transportation. It can be found that *C* ∈ (0,1). Obviously, the larger the C is, the better the coordination state is. Since tourism and transportation are equally important in social development and have equal influence on each other, *α* and *β* are set to 0.5.

Although the coupling between transportation superiority degree and tourism economic development could be expressed by coupling degree, it is not easy to quantify the synergistic effect of transportation and tourism economic development. Under such scenario, this study constructs a coordination degree model of tourism and transportation based on the coupling degree model. So, the degree of coordinated development of transportation and tourism economy, which is defined as (2), could be judged.

(2) $$\begin{array}{c} CC={\left(C\times M\right)}^{1/2}, \end{array}$$

where *CC* is the coupling coordination degree, *C* is the coupling degree, and *M* is the comprehensive harmonization index of transportation and tourism economy, which expresses the overall synergistic effect. *M*=*aT*_1_+*bT*_2_, in which *a* and *b* are undetermined coefficients. There are differences in the degree of mutual promotion and influence between tourism economic and transportation advantage in the coordinated development of transport and tourism. Transportation development will undoubtedly catch up with the process of tourism development, but not vice versa. Tourism development results from the comprehensive action of many factors, and transportation is only one of the driving forces. Therefore, the values of *a* and *b* are assigned according to the actual situation. In this study, *a* and *b* are both set to 0.5.

The coupling coordination degree *CC* integrates the coordination of traffic advantage degree, tourism economic development, and their development level. It has higher stability and wider applicability than a single coordination degree. According to practical application, the value of coupling coordination degree can be roughly divided into four categories [19–21]:

- if 0 < CC ≤ 0.3, *CC* indicate a low coordination coupling
- if 0.3 < CC ≤ 0.5, *CC* shows a moderate coordinated coupling
- if 0.5 < CC ≤ 0.8, *CC* reflects highly coordinated coupling
- if 0.8 < CC < 1, *CC* reflects extremely coordinated coupling

**Figure 2:**
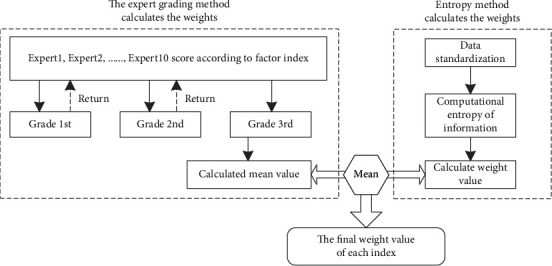
The process of index weight calculation.

In (1), *T*_1_ and *T*_2_, representing comprehensive indexes, are determined based on the selection of index factors and weights. The weights can be calculated using the entropy method [22] and the expert grading method [23]. The calculation process is shown in Figure 2:

Inspired by the geometrical fuzzy approach presented by Versaci et al. [24], we consider that the randomness increases with the uncertainty, the more discrete the data, the greater the information contained, the greater the entropy, the smaller the weight. Therefore, entropy is selected to measure the weight of multiple types of data. The calculation process of entropy method is as follows.

Supposed that there are *n* samples and *m* indicators in the data, where *O*_*ij*_ represents the *j*th indicator of the *i*th sample (1 ≤ *i* ≤ *n*, 1 ≤ *j* ≤ *m*). The information entropy of each indicator is expressed by *E*_*j*_, and the calculation formula is shown as formula (3) and formula (4):

(3) $$\begin{array}{c} E_{j}=-\frac{1}{\text{ln}n}\sum_{i=1}^{n}p_{ij}\cdot {\text{lnp}}_{ij}, \end{array}$$

(4) $$\begin{array}{c} p_{ij}=\frac{O_{ij}^{\prime }}{\sum_{i=1}^{n}O_{ij}^{\prime }}. \end{array}$$

In formula (4), *p*_*ij*_ is the ratio of the standardized index value to the sum of all values of the index attribute.

The entropy *E*_*1*_*, E*_*2*_, ··· *Em* of each index can be calculated by formula (3), and then the weight value of each index can be calculated by entropy method. Formula (5) is as follows:

(5) $$\begin{array}{c} K_{j}=\frac{1-E_{j}}{m-\sum_{j=1}^{m}E_{j}},\;\left(1\leq j\leq m\right). \end{array}$$

Entropy method can clearly reflect the ability to distinguish between indicators, so as to determine the weight. It is an objective weighting method with a certain theoretical basis. Compared with subjective weighting, it has higher reliability and accuracy. The algorithm is relatively simple and can be completed without using professional analysis software. However, entropy method also has some shortcomings and limitations, mainly reflected in the low degree of intelligence. It cannot consider the correlation between indicators. Without the supervision and guidance of business experience, the weight may be distorted.

In view of this, there are disadvantages in calculating the weight of each index only by entropy method, which should be combined with expert scoring method.

The principle of expert grading is illustrated as follows. Firstly, 10 experts in each field are selected to score and evaluate each indicator according to the actual situation. In order to make the expert scoring more refined and accurate, this study adopts three times of reverse-correction scoring, that is, after the first score is summarized, the scores from all experts are returned to each expert for the second score, and so on for three times in total. Finally, the average score of 10 experts is calculated.

### 2.2. Gravity Center Model

The gravity center model is often used to study the spatial structure of the regional economy. The economic center gravity means that the comprehensive strength of a specific subject attribute in the regional economic space can maintain balance in all directions [25]. It can reflect the locational relations and spatial characteristics of the thematic features carried by geographical space and express the spatial clustering trend and aspects of objects and phenomena.

#### 2.2.1. Gravity Center Coordinates

The concept of a gravity center was initially derived from Newtonian mechanics, which refers to a point in a region of space around the forces that are relatively balanced in all directions [26]. The gravity center can dynamically express the magnitude of the force in each secondary area and then move towards the more significant point. The direction of movement depends on the change in the spatial pattern of variables.

Two factors determine the gravity center, including the geographical location of each subregion and the attributes of the selected factors related to the topic. Assuming that a region consists of n subregions, the geographic coordinate of the central city is (*X*_*i*_, *Y*_*i*_), and *W*_*i*_ is the magnitude value of an attribute (or a combination of several attributes) of the subregion, the geographic coordinate of the gravity center of a topic attribute (*X*, *Y*) can be calculated according to(6) and (7),

(6) $$\begin{array}{c} X=\frac{\sum_{i=1}^{n}W_{i}x_{i}}{\sum_{i=1}^{n}W_{i}}, \end{array}$$

(7) $$\begin{array}{c} Y=\frac{\sum_{i=1}^{n}W_{i}y_{i}}{\sum_{i=1}^{n}W_{i}}, \end{array}$$

where *X* and *Y* are gravity center coordinates of a topic in the research area, *X*_*i*_ and *Y*_*i*_ are gravity center coordinates of subregion *i*, *n* is the number of subregion, and *W*_*i*_ is the weight of theme elements in subregion *i*.

To judge the spatial change in the gravity center of a topic, a variety of factors should be considered, that is, *W* in (6) and (7) should be modified. Thus, the gravity center-corrected model can be obtained as (8) and (9),

(8) $$\begin{array}{c} X=\frac{\sum_{i=1}^{n}\left(x_{i}*\sum_{j=1}^{m}\left(w_{j}*k_{j}\right)\right)}{\sum_{i=1}^{n}\sum_{j=1}^{m}\left(w_{j}*k_{j}\right)}, \end{array}$$

(9) $$\begin{array}{c} Y=\frac{\sum_{i=1}^{n}\left(y_{i}*\sum_{j=1}^{m}\left(w_{j}*k_{j}\right)\right)}{\sum_{i=1}^{n}\sum_{j=1}^{m}\left(w_{j}*k_{j}\right)}, \end{array}$$

where *W* represents multiple factors selected around the theme, *j* represents selected index items, *m* represents the total number of selected index items, and *k* represents the weight of each index. The weight *k* value can be determined by entropy and expert scoring methods.

#### 2.2.2. Spatial Coupling Dynamics Based on Gravity Center Offset

The gravity center offset of tourism and transportation can be expressed by moving distance *D*_*ij*_ and moving direction *θ*_*ij*_. *D*_*ij*_ can be calculated according to geographical coordinates (latitude and longitude), which can be defined as ,

(10) $$\begin{array}{c} D_{ij}=R\times \frac{\pi }{180}\times \;\cos^{-1}\left(\sin\;N_{i}\times \;\sin\;N_{j}\times \;\cos\left(E_{i}-E_{j}\right)+\cos\;N_{i}\times \;\cos\;N_{j}\right), \end{array}$$

where *D*_*ij*_ is the distance of gravity center in adjacent years, *R* is the radius of the earth (6371 km), and (*E*_*i*_*, N*_*i*_) and (*E*_*j*_*, N*_*j*_) represent the longitude and latitude geographic coordinates, which represent two adjacent gravity centers in an adjacent year.

The interannual movement direction of the gravity center can be denoted by angle, which can be defined as,

(11) $$\begin{array}{c} \theta_{ij}=\frac{n\pi }{2}+\;\tan^{-1}\left[\frac{N_{i}-N_{j}}{E_{i}-E_{j}}\right], \end{array}$$

where *θ* represents the angle of interannual movement of gravity center. Different value of *θ* represents different directions, which are as follows:

  If *θ*=0°, it represents east direction
  If 0° < *θ* ≤ 90°, it means the gravity center moves to the northeast
  If 90° < *θ* ≤ 180°, it hints that the gravity center moves to the northwest
  If 180° < *θ* ≤ 270°, it means the gravity center moves to the southwest
  If 270° < *θ* ≤ 360°, it indicates that the gravity center moves to the southeast

The spatial coupling dynamics can be evaluated by *S*_*ij*_ and *G*_*ij*_ [27, 28]. *S*_*ij*_ means the spatial overlapping of gravity center offset, and *G*_*ij*_ means the variation consistency of gravity center offset. *S*_*ij*_ can be defined as

(12) $$\begin{array}{c} S_{ij}=\sqrt{{\left(E_{i}-E_{j}\right)}^{2}+{\left(N_{i}-N_{j}\right)}^{2}}, \end{array}$$

where (*E*_*i*_*, N*_*i*_) and (*E*_*j*_*, N*_*j*_) represent gravity center coordinates of tourism and transportation in the same year. *S*_ij_ represents the spatial distance. Its value represents the level of coupling coordination degree. *G*_*ij*_ can be defined as Equation (13),

(13) $$\begin{array}{c} G_{ij}=\cos\left(\tan^{-1}\left(\frac{\left(N_{i}-N_{i-1}\right)}{\left(E_{i}-E_{i-1}\right)}\right)-\;\tan^{-1}\left(\frac{\left(N_{j}-N_{j-1}\right)}{\left(E_{j}-E_{j-1}\right)}\right)\right), \end{array}$$

where (*E*_*i*_*, N*_*i*_), (*E*_*i-1*_*, N*_*i-1*_), (*E*_*j*_*, N*_*j*_), and (*E*_*j-1*_*, N*_*j-1*_) represent the gravity center coordinates of tourism and transportation in two adjacent years. *G*_*ij*_ represents the cosine of the included angle of two vectors. Its value represents the level of anomalous consistency.

### 2.3. Spatial Coincidence Degree

To further represent the overlapping degree of different spatial entities in the same period, this study proposed the concept of spatial coincidence degree, which indicates the association between various spatial entities. The greater the spatial coincidence degree, the stronger the association is. According to the spatial object distribution of different spatial entities, this article further defines spatial coincidence degree based on clustering and spatial coincidence degree calculation based on clustering.

#### 2.3.1. Spatial Coincidence Degree Calculation Based on Clustering

**Figure 3:**
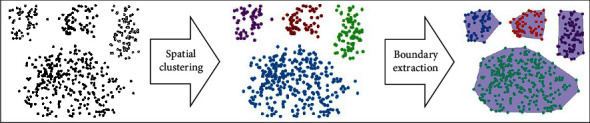
Spatial clustering process of point ground objects. The black dots in the left panel represent the distribution before processing. After spatial clustering, the distribution of points is divided into different color divisions in the middle panel. The figure in the right panel is the state of boundary extraction for spatial clustering.

This article proposed a spatial coincidence degree calculation based on clustering for the spatial entities with dense distribution and prominent clustering characteristics. Spatial clustering of two spatial entities is first conducted to form their respective clustering polygons [29]. The calculation process is shown in Figure 3:

This calculation process can be defined as

(14) $$\begin{array}{c} SC=\frac{\left(\sum_{i=1}^{n}P_{i}\right)\left(\sum_{j=1}^{m}Q_{j}\right)}{\left(\left[\left(\sum_{i=1}^{n}P_{i}\right)\cup \left(\sum_{j=1}^{m}Q_{j}\right)\right]/2\right)}, \end{array}$$

where *P* and *Q* represent spatial entities and *W* represents the coincidence degree of them. *P*_*i*_ defines the area after *P* clustering. *i* represents the secondary classification index subordinate to *P*. The area after clustering of *Q* could be represented by *Q*_*j*_. *j* represents the secondary classification index subordinate to *Q*.

#### 2.3.2. Spatial Coincidence Degree Calculation Based on Buffering

There is an apparent overlap between the two types of spatial features in the case of sparse distribution. Therefore, a buffering-based spatial coincidence calculation method is proposed in this article. The method first needs to establish buffers for the spatial features. According to the subordinate secondary indicators of each type of ground feature, buffer radius can be set differently. Then, we can obtain the neighborhood spatial coincidence degree, which is defined as (15), after calculating the same kind of buffer and the neighborhood range of space entities.

(15) $$\begin{array}{c} SC=\frac{M\left(\sum_{i=1}^{n}P_{i}\right)\cap M\left(\sum_{j=1}^{m}Q_{j}\right)}{\left(\left[M\left(\sum_{i=1}^{n}P_{i}\right)\cap M\left(\sum_{j=1}^{m}Q_{j}\right)\right]/2\right)}, \end{array}$$

where *SC* represents the spatial coincidence degree between the entity *P* and *Q*. *P*_*i*_ represents the neighborhood of *P*. The secondary classification index subordinate to the entity *P* is represented by &ImaginaryI;. *Q*_*j*_ means the neighborhood of *Q*. *j* represents the secondary classification index subordinate to *Q*. *M* indicates the neighborhood fusion calculation.

In this study, the values of coincidence degree are divided into the following four categories:

- If 0＜ *SC* ≤0.3, *SC* represents a low coincidence degree
- If 0.3＜ *SC* ≤0.5, *SC* indicates a moderate coincidence degree
- If 0.5＜ *SC* ≤0.8, *SC* indicates a high coincidence degree
- If 0.8＜ *SC* ≤1, *SC* represents an extremely high coincidence degree

## 3. Experimental Results and Analysis

### 3.1. Study Area

The study area in this article is Jiangxi Province, which is located in the southeast of China (113°34 ′36 ″-118°28′ 58″ *E*, 24°29 ′14 ″30°04′ 41″ N). It is situated in the southern bank of the middle and lower reaches of the Yangtze River, belonging to eastern China. It borders Zhejiang and Fujian to the east, Guangdong to the south, Hunan to the west, and Hubei and Anhui to the north. The province contains 11 prefecture-level cities and has a superior location and convenient transportation. At the same time, Jiangxi, as an old revolutionary base area in China, is a tourist attraction where the spatiotemporal association between tourism and transportation could be well reflected. Therefore, we choose Jiangxi Province as a representative study area.

### 3.2. Experimental Data

#### 3.2.1. Tourism-Related Data

The article takes scenic spots above the 3 A level in Jiangxi Province as the research object. Tourism data are mainly obtained from the official website of Jiangxi Provincial Department of Culture and Tourism [30] and Jiangxi Provincial Statistical Yearbook [31]. They generally include the number of domestic tourists, the cost of domestic tourists, the number of foreign-related tourists, and foreign tourism receipts collected from foreign-related tourism. Data in prefecture-level cities are received from 2005 to 2019.

It is noted that the geographic spatial position of tourist attractions generally does not change. However, their attributes, including the income, the number of tourists, and the rating, will vary with time. For example, Sanqingshan Scenic Area in Jiangxi Province was a 4 A-level scenic spot before 2011 and was later rated as a 5 A-level scenic spot.

#### 3.2.2. Traffic-Related Data

The traffic-related data mainly include the GPS road data and the POI data of railway stations and airports. The GPS road data in 2005, 2008, 2010, 2013, 2015, 2017, and 2019 are provided by the Jiangxi Surveying and Mapping Institute of China. In addition, the POI data of 74 railway stations and seven airports in Jiangxi Province, as well as the related attribute information such as the number of arrivals of highway mileage at the end of each year in various cities in Jiangxi Province, are also obtained. The information contained in the GPS road network is shown in Table 1:

According to the data collection situation, this study selected high-speed roads, national roads, provincial roads, county roads, high-speed entrances and exits, railway stations, and airports in the transportation network for analysis and research.

### 3.3. The Determination of Index Weight

In this article, the indicators representing the development of tourism include the number of domestic tourists (*X*_1_), the cost of domestic tourists (*X*_2_), the number of foreign-related tourists (*X*_3_), and foreign tourism receipts (*X*_4_). The indicators representing the development of transportation include the number of highway mileages at the end of the year (*Y*_1_), the number of expressway entrances and exits (*Y*_2_), the number of railway stations (*Y*_3_), the number of passenger stations (*Y*_4_), and the passenger aircraft throughput (*Y*_5_) [32–34]. All these data are selected from 2005 to 2019.

We calculate the weights of tourism and transportation indicators according to the entropy method and expert scoring principle and use the arithmetic average as the total index weights.  Table 2 The calculation results are as follows:

### 3.4. Coupling Coordination Degree

The coupling degree (*C*) and the coupling coordination degree (*CC*) are calculated using the analysis method of coupling coordination degree between tourism and transportation as defined as (1) and (2) and are shown in Table 3. The coupling coordination degree of tourism and transportation in Jiujiang, Shangrao, Ji'an, and Ganzhou is relatively high and stable, with an average value between 0.83 and 0.91. Meanwhile, the coupling degree of Xinyu and Jingdezhen changes significantly, with a minimum value of only 0.10. Jiujiang has the highest and most stable coupling coordination degree, with a mean value of 0.84. The coupling coordination degree of Shangrao increases yearly with an average annual growth of 0.13. Yingtan and Xinyu have the lowest coupling coordination degree, and there is no apparent rising trend.

**Figure 4:**
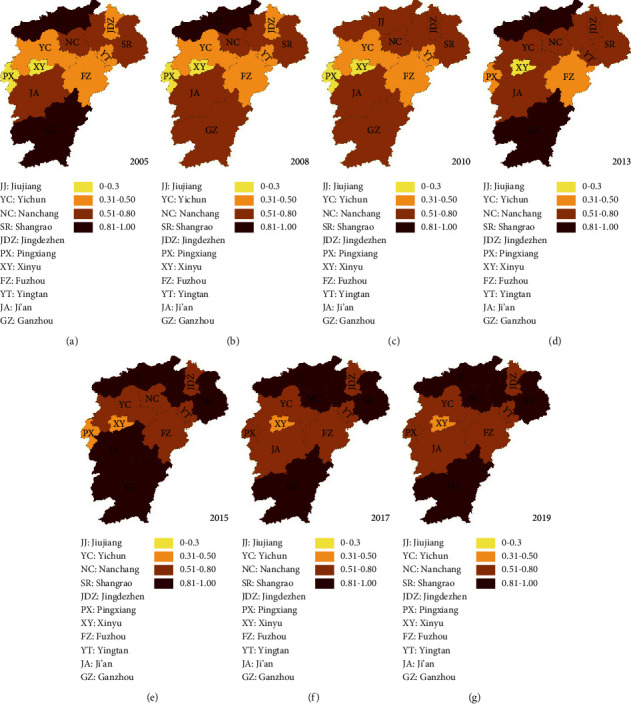
Coupling coordination degree of different cities from 2005 to 2019. According to the results of coupling coordination degree, it can be divided into four categories: 0–0.3 is low coordination coupling, 0.3–0.5 is moderate coordination coupling, 0.5–0.8 is high coordination coupling, and 0.8–1 is extremely coordination coupling, which are represented by light yellow, orange, brown, and dark red, respectively.

The study applies ArcGIS10.2 software to select the data in four years (2005, 2010, 2015, and 2019) and clearly exhibits the coupling coordination degree of tourism and transportation among the 11 cities. The thematic maps are generated and shown in Figure 4. The coupling coordination degree of tourism and transportation shows a positive trend. Until 2019, there are eight cities with high coupling coordination degrees in the study area. However, significant differences between regions exist. Jiujiang city has maintained a high coupling coordination degree in 15 years. Xinyu city shows a relatively stable trend but a low coupling coordination degree. The coupling coordination degrees of Nanchang and Shangrao had noticeably changed from a moderate level before 2010 to a very high level.

### 3.5. Gravity Model Calculation

This study initially applies the gravity model to analyze the regional structure of Jiangxi Province, including its foreign-related inbound tourism and domestic tourist market. According to the model as defined in Equations (6) and (7), the gravity center of inbound tourist flow and reception of domestic tourists can be calculated as shown in Table 4 by using the number of inbound tourists and domestic tourists received in Jiangxi Province from 2005 to 2019. From Table 4, it can be found that the gravity center of the inbound tourist flow of Jiangxi Province from 2005 to 2019 falls in Nanchang city, while the gravity center of domestic tourists falls in Yichun city.

### 3.6. The Improved Gravity Center Model and Calculation of Spatial–Temporal Characteristics of Migration

When the weights of indicators have been calculated, we can calculate the gravity center coordinates of tourism and transportation in Jiangxi Province from 2005 to 2019 based on the improved gravity center model formulas (8) and (9). The annual offset distance and moving direction can be obtained according to (10) and (11). The calculation results are shown in Table 5. The gravity center of Jiangxi province tourism development dispersed in the range of 115.8957°E ∼ 116.1163°E, 28.1818°N ∼ 28.4696°N during 2005–2019, which mainly located in the southwest of Nanchang city and the east of Fengcheng in Yichun city. The process of the gravity center migration can be divided into two stages. From 2005 to 2010, the tourism center shifted to the northeast year by year, basically along the border area of Yichun and Nanchang. From 2011 to 2019, the tourism center gradually turned to the southwest and circled in the junction area of Jinxian County in Nanchang and Fengcheng in Yichun.

According to (12) and (13), spatial overlap and variation consistency parameters can be calculated as shown in Table 6. The spatial overlap of the gravity center of tourism and transportation in Jiangxi Province tends to be stable with time passing by. The spatial distance of tourism and transportation decreases from 99.38 km to 74.17 km, and their gravity center tends to be closer. The interannual anomalous consistency of the gravity center of tourism and transportation is not balanced, ranging from 0.0545 to 0.9924.

### 3.7. Spatial Coincidence Degree

Although the spatial distribution of research objects is sparse, there is obvious overlap in macro. Thus, our study adopts the buffer to calculate spatial coincidence degree. In the light of the 5 A, 4 A, and 3 A scenic spot classification, the buffer radius can be set to 50 km, 40 km, and 30 km, respectively. In this case, 353 buffer zones are established. In the transportation network, the buffer radii of airports, high-speed railway stations, and ordinary railway stations are set to 30 km, 20 km, and 15 km, respectively. And 81 buffer zones are established then. As for expressway, national highway, provincial highway, and county highway, the cluster radius is set to 25 km, 20 km, 15 km, and 10 km, respectively. A series of line-cluster areas are established.  Table 7 integrates the buffer settings:

According to (15), the spatial coincidence degree of tourist attractions and traffic network in Jiangxi province from 2005 to 2019 is calculated and shown in Table 8. The spatial coincidence degree change of 353 tourist attractions above the 3 A level and transportation network in Jiangxi Province presents an upward trend with time. The moderate spatial coincidence degree in 2005 had risen to an extreme level (0.81) in 2019.

This study also calculates the spatial coincidence of different years in different cities, as shown in Figure 7. The spatial coincidence of different areas varies from each other. Nanchang (0.83) has the highest spatial coincidence degree between transportation and tourism, followed by Jiujiang (0.82). The spatial coincidence degree of Yingtan city is the lowest, indicating a weak coincidence state.

## 4. Discussion

### 4.1. Analysis of Coupling Coordination Degree between Tourism and Transportation

**Figure 5:**
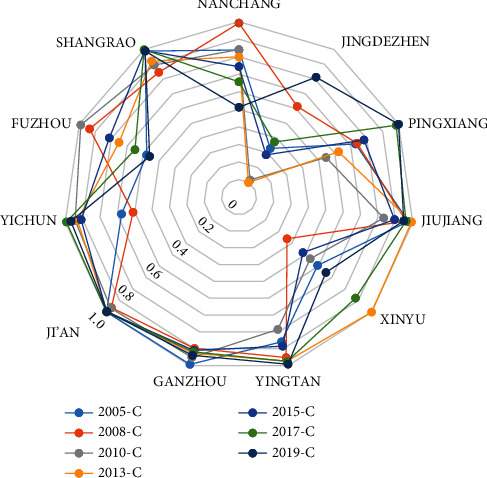
Coupling degree of tourism and transportation in different cities of Jiangxi Province from 2005 to 2019.

**Figure 6:**
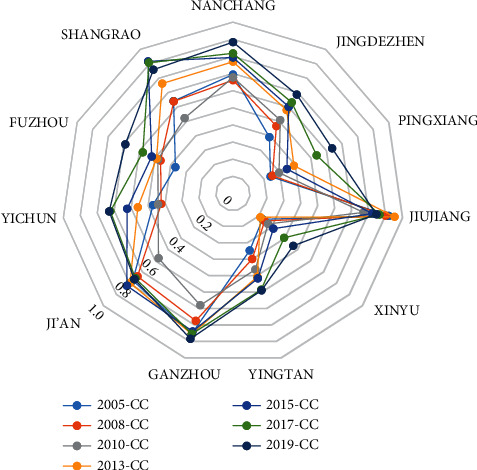
Coupling coordination degree of tourism and transportation in different cities of Jiangxi Province during 2005–2019.

**Figure 7:**
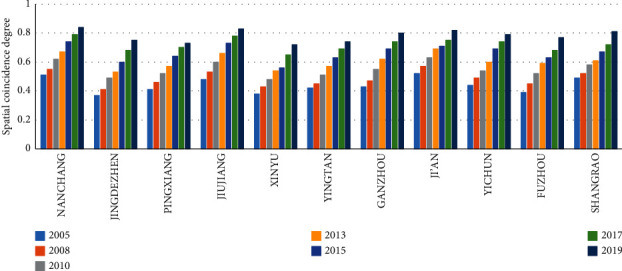
Spatial coincidence degree of tourist attractions and transportation network in Jiangxi Province from 2005 to 2019.

Figures 5 and 6 coupling degree and coupling coordination degree of the transportation development and tourism economy of Jiangxi province generally show an upward trend over time. Economically speaking, the development of transportation has promoted the growth of the tourism economy. However, the coupling coordination degree of local areas is not balanced, showing a good, medium, and poor band distribution. In 2019, the coupling coordination degrees of tourism and transportation in Jiujiang city, Shangrao city, and Ganzhou city exceeded 0.8. The degree of Jiujiang city was the highest and stable, closely related to the construction of the Beijing–Kowloon railway, the layout of airports and docks, and the addition of expressways. The coupling coordination degree between Yingtan and Xinyu is low, while other cities' degree are medium or above.

### 4.2. Changes in the Gravity Center of Tourist Number Receiving Domestic and Foreign Tourists

#### 4.2.1. The Characteristics for Changes in the Gravity Center of Foreign Tourist Number

**Figure 8:**
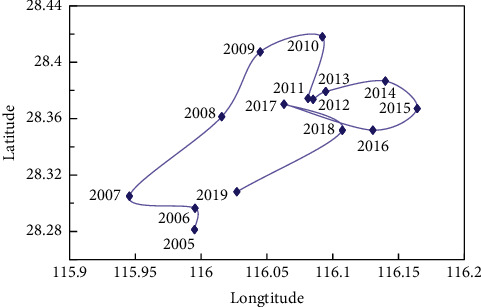
Change trajectory of gravity center of tourist number receiving foreign tourists in Jiangxi Province from 2005 to 2019.

Figure 8 shows the changes in the gravity center of inbound tourists in 11 cities of Jiangxi Province from 2005 to 2019. The gravity center of tourist numbers has changed apparently since 2005. It gradually moved northeast from the junction of Nanchang and Yichun city from 2005 to 2012 and moved from Nanchang city to the southwest during 2012–2019.

#### 4.2.2. The Change in Gravity Center of Domestic Tourist Number

**Figure 9:**
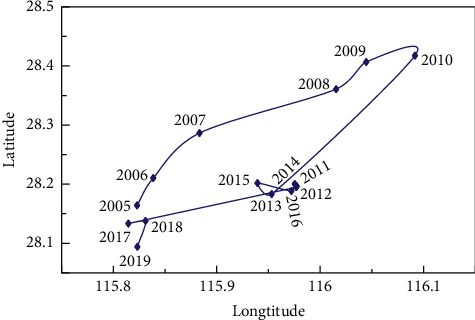
The migration trajectory of gravity center of domestic tourist number in Jiangxi Province from 2005 to 2019.

The gravity center of the number of domestic tourists received by 11 cities in Jiangxi province from 2005 to 2019 is shown in Figure 9, which had changed evidently. From 2005 to 2010, the gravity center gradually shifted from Yichun to the north. During 2010–2019, the gravity center gradually shifted south from the Nanchang city border to Yichun city.

According to the data analysis results mentioned above, the center of gravity of domestic tourists in Jiangxi province fluctuates around Fengcheng city, and the center of gravity of foreign tourists in Jiangxi Province almost fluctuates around the Xinjian district of Nanchang City. It demonstrates that the purposes or strategies of domestic and foreign tourists to Jiangxi are different.

### 4.3. Offset and Direction of Gravity Center of Tourism and Transportation

As shown in Table 5, the maximum annual offset of the tourism gravity center is 25.97 km, while the minimum annual offset is 2.17 km. The total offset in 15 years is 104.36 km. From 2005 to 2011, the annual offset had changed considerably, with an average of 10.57 km. From 2012 to 2018, the annual offset was relatively stable, with an average of 3.98 km. In 2019, the annual offset peaked again, reached to 13.09 km.

**Figure 10:**
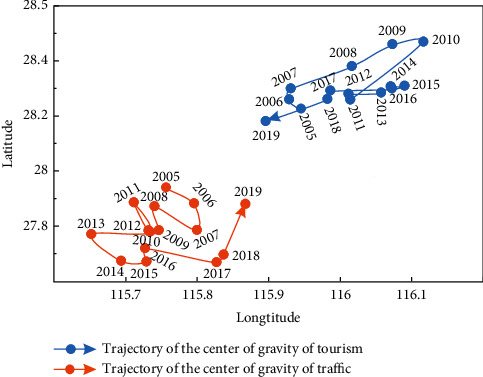
Gravity center movement trajectory of tourism and transportation in Jiangxi Province from 2005 to 2019.

According to the moving direction data, the tourism gravity center has mainly shifted northeast from 2005 to 2015 (Figure 10). It indicates that the provincial capital Nanchang has significantly promoted tourism. From 2016 to 2019, the gravity center moved to the southwest yearly. Tourism in Yichun city has developed rapidly since 2016 and has formed an aggregation effect. In general, compared with the geometric center (116.03°E, 27.28°N), the tourism center has been rotating in the northeast direction.

During 2005–2019, the gravity center of transportation is distributed in the range of 115.5673°E ∼ 116.6562°E, 27.5812°N ∼ 27.6868°N, mainly located in the junction of Xingan County in Yichun city and Lean County in Fuzhou city. Overall, the gravity center of transportation in Jiangxi Province moves to the northwest first and then gradually to the southwest.

According to the gravity center offset data of transportation (Table 5), the maximum annual offset is 11.64 km, while the minimum annual offset is 0.11 km. The total offset in 15 years is 33.16 km. From 2005 to 2010, the annual offset had significantly changed, with an average of 3.59 km. During 2011–2018, the annual offset was relatively stable, with an average of 0.45 km. The annual offset peak appeared again in 2019.

As shown in Figure 10, the gravity center of transportation mainly moved northwest and southeast during 2005–2014. From 2015 to 2019, the expressways in Yichun and Ji'an cities had developed rapidly, driving the transport of the whole province. Thus, the gravity center had gradually and significantly shifted to the southwest. Overall, the gravity center of transportation is very close to the geometric center of Jiangxi Province (116.03°E, 27.28°N) with an average distance of only 78.53 km, indicating that the spatial difference of the transportation network in Jiangxi Province is slight.

### 4.4. Analysis of Spatial Coincidence Degree between Tourism and Transportation

The spatial coincidence degree variation of tourism and transportation during the study period demonstrates that tourist attractions will have grade *A* change with time. As a result, the buffer radius and the buffer area will increase. The development trend and buffer area of transportation are growing. The spatial coincidence degree of tourism and transportation in Jiangxi Province is also increasing, but the growth rate is not.

### 4.5. Comprehensive Analysis of CGS Spatiotemporal Association between Tourism and Transportation in Jiangxi Province

In this article, we use the gravity center model method, coupling coordination model, and spatial coincidence algorithm to check whether there is consistency.

**Figure 11:**
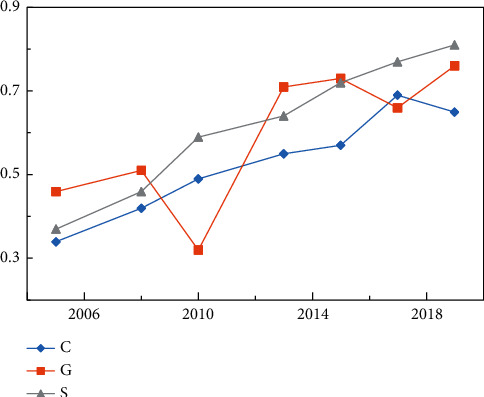
Comparison of tourism and transportation correlation results based on CGS model.

Figure 11 shows the association between tourism and transportation in Jiangxi Province from 2005 to 2019 based on the CGS model. It can be found that the coupling coordination degree, the consistency of gravity offset, and the spatial coincidence degree of tourism and transportation in Jiangxi Province all show a gradual upward trend. Especially the upward trends in coupling coordination degree and gravity center degree are almost the same (the consistency is 83.6%), which shows that the spatial layout of tourism and transportation is directly related to their development. The change in the gravity center of tourism and transport has a certain degree of fluctuation. Policy, population, local economy, and others are external influences.

The data analysis results of the three models can be expressed as linear functions, and the evolutionary trend functions of coupling coordination degree, gravity center model, and spatial coincidence degree are as follows:

(16) $$\begin{array}{c} Y_{C}=\;0.0239X-47.620\left(R^{2}=0.9461\right),\\ Y_{G}=\;0.0253X-50.362\left(R^{2}=0.5933\right),\\ Y_{S}=\;0.0319X-63.597\left(R^{2}=0.9782\right). \end{array}$$

where *R*^*2*^ represents the determination coefficient, which is an index measuring the fitting degree of the trend line and can reflect the fitting degree between the estimated value of the trend line and the corresponding actual data. The slope of the trend function of the three models shows strong similarity and their standard deviation is only 0.000018, which further illustrates the applicability and validity of the selected models.

## 5. Conclusion

This study selects 11 cities in Jiangxi Province as the research object and proposes a CGS spatiotemporal association model using the coupling coordination model, gravity center model, and spatial coincidence algorithm. Overall, the study has the following contributions:

1. The indexes of coupling coordination degree are calculated more accurately. The weight of each index is determined by entropy and expert scoring methods, which calculates the coupling coordination index more scientifically and precisely. The coupling coordination degree of tourism and transportation in Jiangxi province gradually increases from 2005 to 2019, but becomes worse in 2010, with no cities in the state of extremely high coupling coordination. The highest appears in 2013, with four cities having extremely high coupling coordination degree; however, if calculated according to the provincial average, the highest would be 0.73 that appears in 2019.
2. Improving gravity center model algorithm. In traditional gravity center model, *W* is the topic magnitude value. The gravity center coordinates (*X, Y*) can reflect the spatial position of the topic at a specific time. In the case of a multi-attribute subject, *W* needs to be modified by introducing the weight *k* to obtain the modified gravity coordinates (*X*′,  *Y*′) and to calculate the gravity center of migration distance *D.* Finally, the spatial coupling dynamic characteristics of the gravity center between tourism and transportation can be obtained from spatial overlap *S* and variation consistency *G*. The spatial overlap of the centers of gravity of tourism and transportation in Jiangxi province gradually tends to be stable, and the spatial distance is shortened from 99.38 km to 74.17 km, but the interannual variability of the center of gravity is not balanced, from 0.9924 to 0.0545.
3. Proposing the concept and calculation method of spatial coincidence degree. According to the spatial distribution characteristics of ground objects, two kinds of methods are defined to calculate the spatial coincidence degree. The first method is the space alignment algorithm based on clustering analysis. The second calculation method is based on the analysis of the cluster space alignment algorithm. The spatial coincidence between tourist attractions and traffic network changed from moderate in 2005 to extreme in 2019, with the coincidence degree reaching 0.81. The spatial coincidence degree of Nanchang is the highest (0.83) and Yingtan's degree is the lowest (weak coincidence state).
4. The gravity center model, coupling coordination model, and spatial coincidence degree are combined for analysis. Based on the dynamic coupling analysis of the gravity center migration of tourism and transportation, the migration characteristics of the gravity center of tourism and transportation in Jiangxi Province, as well as their spatial overlap and consistency of changes, are obtained. Through applying the coupling degree and coupling coordination degree algorithm, the coupling coordination between tourism economic development and transportation network development in Jiangxi Province is obtained and expressed quantitatively. By using the spatial matching algorithm, the spatial matching characteristics of tourist attractions and transportation networks in Jiangxi Province are also obtained. Finally, the correlation analysis on the results of three aspects is carried out. The overall tourism development in Jiangxi Province and the development of transportation shows a positive trend for mutual promotion and mutual restriction, while regional differences also exist. The travel and transportation center of gravity of Nanchang city has a strong consistency variation. Meanwhile, the coupling coordination degree is high, and the space alignment also exhibits a high state. However, tourism and transportation in Xinyu have a poor overlap of the gravity center, a moderate coupling coordination degree, and a slow growth of spatial coincidence degree.

This study utilizes various data and combines three model methods to explore and mine the spatiotemporal correlation information between tourism and transportation. However, it is impossible to apply a clustering method when calculating the spatial coincidence degree due to the small amount of point data in the research area. In the future, we will carry out analysis and research from the multiscale of time and space and try to find association rules between tourism and transportation. By comparing the association rules obtained by various methods, we will try to provide a solid support for improving the harmonious development between tourism and transportation.

## Figures and Tables

**Table 1:** Transportation network data information of Jiangxi Province (2019).

No.	Data name	Type	Number
1	High-speed entrances and exits	Point	1137
2	Bridges	Point	13899
3	Toll stations	Point	334
4	High-speed roads	Line	2243
5	National roads	Line	3652
6	Provincial roads	Line	5053
7	County roads	Line	10515
8	Township roads	Line	27817
9	Village roads	Line	159551

**Table 2:** Tourism and traffic index weight calculated by three methods.

Associated factors	Index name	The entropy method	Expert scoring method	Comprehensive index weight
Tourism (X)	Domestic tourism number *X*_1_	0.23	0.33	0.28
	Domestic tourism income *X*_2_	0.27	0.31	0.29
	Number of inbound tourists *X*_3_	0.24	0.19	0.215
	Inbound tourism revenue *X*_4_	0.26	0.17	0.215
Transportation (Y)	Highway mileages at the end of the year, *Y*_1_	0.19	0.37	0.28
	Number of entrances and exits of expressways *Y*_2_	0.21	0.19	0.20
	Number of railway stations *Y*_3_	0.17	0.23	0.20
	Number of passenger stations *Y*_4_	0.23	0.09	0.16
	Passenger aircraft throughput *Y*_5_	0.20	0.12	0.16

**Table 3:** Coupling degree (*C*) and coupling coordination degree (*CC*) of tourism and transportation in different cities of Jiangxi Province from 2005 to 2019.

Area	2005		2008		2010		2013		2015		2017		2019	
	*C*	*CC*	*C*	*CC*	*C*	*CC*	*C*	*CC*	*C*	*CC*	*C*	*CC*	*C*	*CC*
Nanchang	0.84	0.69	0.99	0.66	0.84	0.48	0.8	0.67	0.74	0.65	0.66	0.62	0.51	0.53
Jingdezhen	0.33	0.29	0.61	0.47	0.12	0.21	0.1	0.18	0.29	0.3	0.37	0.33	0.81	0.35
Pingxiang	0.73	0.24	0.74	0.25	0.54	0.19	0.62	0.29	0.78	0.35	0.98	0.54	1	0.39
Jiujiang	0.97	0.92	0.96	0.9	0.83	0.77	0.99	0.95	0.9	0.82	0.96	0.86	0.95	0.84
Xinyu	0.59	0.23	0.36	0.24	0.54	0.27	1	0.21	0.48	0.31	0.88	0.39	0.66	0.46
Yingtan	0.86	0.35	0.95	0.4	0.79	0.26	0.97	0.34	0.88	0.32	0.98	0.28	0.99	0.29
Ganzhou	0.99	0.85	0.9	0.77	0.95	0.68	0.93	0.85	0.91	0.84	0.92	0.85	0.94	0.88
Ji 'an	1	0.77	0.96	0.74	0.97	0.58	1	0.8	1	0.82	1	0.75	1	0.76
Yichun	0.68	0.47	0.61	0.42	0.94	0.44	0.93	0.56	0.91	0.62	0.99	0.72	0.97	0.73
Fuzhou	0.58	0.37	0.94	0.47	0.99	0.38	0.75	0.48	0.81	0.47	0.65	0.48	0.56	0.39
Shangrao	0.99	0.64	0.85	0.64	0.89	0.52	0.92	0.76	0.99	0.92	1	0.91	0.99	0.86

**Table 4:** Gravity center coordinates of receiving overseas and domestic tourists in Jiangxi Province from 2005 to 2019. Visual renderings are shown in Figures 5and 6.

Year	The number of foreign-related tourists		The number of domestic tourists	
	Longitude	Latitude	Longitude	Latitude
**2005**	115.9950	28.2813	115.8225	28.1645
**2006**	115.9954	28.2965	115.8382	28.2108
**2007**	115.9455	28.3050	115.8831	28.2870
**2008**	116.0157	28.3614	116.0157	28.3614
**2009**	116.0449	28.4074	116.0449	28.4074
**2010**	116.0921	28.4181	116.0921	28.4181
**2011**	116.0812	28.3744	115.9531	28.1836
**2012**	116.0851	28.3737	115.9394	28.2022
**2013**	116.0947	28.3792	115.9723	28.1889
**2014**	116.1400	28.3867	115.9755	28.2007
**2015**	116.1642	28.3672	115.9774	28.1971
**2016**	116.1306	28.3518	115.9770	28.1952
**2017**	116.0630	28.3702	115.8141	28.1337
**2018**	116.1074	28.3518	115.8307	28.1383
**2019**	116.0272	28.3081	115.8228	28.0944

**Table 5:** Gravity center offset and direction of tourism and transportation.

Year	Tourism				Transportation			
	Gravity center longitude	Gravity center latitude	Offset/km	Direction	Gravity center longitude	Gravity center latitude	Offset/km	Direction
2005	115.9451	28.2269	—	—	115.6562	27.6410	—	—
2006	115.9285	28.2606	4.1725	Northwest	115.6456	27.6830	4.8163	Northwest
2007	115.9307	28.3001	4.3929	Northeast	115.5995	27.6866	5.1403	Northwest
2008	116.0162	28.3814	13.1172	Northeast	115.6402	27.6730	4.7696	Southeast
2009	116.0729	28.4607	10.8324	Northeast	115.6462	27.6860	1.5943	Northeast
2010	116.1163	28.4696	4.9187	Northeast	115.6317	27.6848	1.6252	Southwest
2011	116.0139	28.2595	25.9744	Southwest	115.6313	27.6868	0.2204	Northwest
2012	116.0114	28.2811	2.4243	Northwest	115.6336	27.6800	0.7970	Southeast
2013	116.0571	28.2855	5.1087	Northeast	115.6318	27.6716	0.9564	Southeast
2014	116.0706	28.3076	2.8764	Northeast	115.6335	27.6748	0.4088	Northeast
2015	116.0900	28.3097	2.1659	Northeast	115.6289	27.6719	0.6072	Southwest
2016	116.0722	28.3000	2.2505	Southwest	115.6270	27.6701	0.2919	Southwest
2017	115.9861	28.2926	9.5982	Southwest	115.6269	27.6691	0.1058	Southwest
2018	115.9820	28.2619	3.4372	Southwest	115.6268	27.6674	0.1907	Southwest
2019	115.8957	28.1818	13.0864	Southwest	115.5673	27.5812	11.6355	Southwest

**Table 6:** Spatial coupling characteristics of gravity center of tourism and transportation.

Year	Spatial overlap/km	Anomalous consistency
2005	71.0619	—
2006	69.9770	0.4634
2007	75.5645	0.0546
2008	86.9968	0.6134
2009	95.7836	0.8021
2010	99.3778	0.9924
2011	73.9358	0.1198
2012	76.4479	0.1726
2013	80.0283	0.2170
2014	82.4166	0.9648
2015	84.1411	0.8866
2016	82.5692	0.9231
2017	77.7812	0.0545
2018	74.7460	0.2547
2019	74.1677	0.8887

**Table 7:** Buffer radius settings of scenic spots and transportation network.

Category	Type	Cluster base	Buffer radius/km
Tourist attractions	Dot	5 A scenic spots	50
		4 A scenic spots	40
		3 A scenic spots	30
Transportation network	Dot	Airport	30
		High-speed station	20
		General station	15
	Linear	Expressway	25
		National highway	20
		Provincial highway	15
		County highway	10

**Table 8:** Spatial coincidence degree of tourist attractions and transportation network in Jiangxi Province from 2005 to 2019.

Year	Coincidence S	Qualitative
2005	0.47	Moderate
2008	0.56	Moderate
2010	0.59	Highly
2013	0.64	Highly
2015	0.72	Highly
2017	0.77	Highly
2019	0.81	Extreme

## Data Availability

The data used to support the findings of the study are available from the corresponding author upon request.
